# Patterned Metal Flexible Films as a Strain Sensor with Good Durability and Anti-Corrosion Property

**DOI:** 10.3390/mi17040464

**Published:** 2026-04-11

**Authors:** Xu Zheng, Qing Wang, Wenming Cao, Wenchao Li, Rui Zhang, Ping Xiang, Yijia Liu

**Affiliations:** 1 School of Civil and Architectural Engineering, Taishan University, Taian 271000, China; zhengxu@sdu.edu.cn (X.Z.);; 2 Changjiang Water Conservancy and Hydropower Engineering Construction (Wuhan) Co., Ltd., Jinan 250109, China; 3 Shandong Engineering Research Center of High-Durability and Corrosion-Resistant New Building Materials, Taishan University, Taian 271000, China

**Keywords:** strain sensor, patterned sandwich structure, anti-corrosion property, high durability

## Abstract

To prevent corrosion in humid environments and electrical failure under loads, we developed a highly durable corrosion-resistant flexible strain sensor with a patterned sandwich structure. The effects of film dimensions and ambient temperature on the sensor’s electrical conductivity were investigated separately. The patterned flexible strain sensor demonstrated exceptional durability, maintaining stability after multiple tensile cycles and large deformations. The PDMS coating effectively protected the conductive layer from external environmental factors. Experimental results revealed that the sensor could efficiently block the corrosive effects of humid environments. Furthermore, when applied to real-time micro-strain detection in steel plate tensile tests, the relationship between Δ*R/R*_0_ and strain exhibited high linearity and sensitivity. The conductive film shows excellent durability and corrosion resistance, demonstrating significant application potential as a flexible strain sensor in humid conditions.

## 1. Introduction

Structural health monitoring (SHM) technology has emerged as a critical tool for engineering management and maintenance [1,2,3]. Long-term structural monitoring enables real-time health assessment, facilitating proactive maintenance strategies to enhance structural reliability, prolong service life, and prevent catastrophic failures, which is of vital practical significance for modern infrastructure, wearable health monitoring and intelligent human–machine interaction systems [4,5,6]. Among various SHM and flexible sensing techniques, resistive strain sensors hold particular promise and have become an indispensable core component due to their direct mechanical-electrical coupling characteristics, simple preparation process and easy signal acquisition [6,7,8]. In recent years, a variety of novel flexible strain-sensing systems have been developed to meet the needs of diversified application scenarios: helical ionotropic gel fiber sensors have achieved omnidirectional strain perception through topological structure design, which provides a new solution for multidimensional motion monitoring [9]; MXene-based flexible sensors have shown excellent application potential in wearable electronics owing to their high metallic conductivity and mechanical flexibility [10]; and stretchable organic/inorganic hybrid electrodes have also been widely used in self-powered wearable sensing systems, which further expands the application boundary of flexible strain sensors [11]. However, the existing sensing systems still face common bottlenecks in practical engineering applications, especially in the long-term service under harsh outdoor and corrosive environments, which puts forward higher requirements for the mechanical durability, environmental stability and batch reproducibility of strain-sensing materials.

Current commercial resistive strain-sensing materials and mainstream novel sensing systems exhibit significant limitations in both environmental stability, mechanical durability and batch reproducibility, which severely restrict their long-term service in practical engineering. Traditional surface mount strain gauges are highly susceptible to environmental degradation caused by prolonged exposure to humidity, salt spray, and industrial pollutants. As a result, their operational lifespan is substantially reduced under corrosive conditions, which severely restricts their applicability in harsh or outdoor environments. Although smooth metal film sensors offer advantages in terms of mass production and process consistency, their conductive layers are prone to cracking under mechanical deformation. This failure arises from strain localization and necking effects, which compromise sensor functionality—particularly under dynamic loading conditions where repeated strain can lead to rapid performance degradation [12,13]. To address this issue, existing studies have tried to suppress crack propagation of metal films through micro/nano convex structure design, or regulate crack evolution via substrate surface modification to balance the sensitivity and stretchability of sensors [14,15]. However, these strategies are still based on continuous metal film systems, which cannot fundamentally eliminate the strain localization effect of the film under deformation, and it is still difficult to avoid the irreversible conductivity degradation caused by crack propagation under long-term cyclic loading and large deformation. Meanwhile, the lack of targeted anti-corrosion design makes it difficult to meet the demand of long-term service in harsh environments.

Metal nanowires have emerged as a promising alternative due to their high aspect ratio, excellent electrical conductivity, and mechanical flexibility. Under tensile or bending strain, these nanowires can interconnect to form conductive pathways, effectively mitigating the conductivity loss typically observed in continuous metal films caused by crack propagation [16,17,18]. To further optimize the sensing performance, some studies have realized the regulation of strain distribution through strain-engineered stretchable substrate, so as to balance the sensitivity and stretchability of silver nanowire composite sensors [19]. However, despite these optimization strategies, the random distribution of nanowires within existing thin-film composites introduces significant reproducibility issues. This stochastic nature not only leads to inconsistent electromechanical performance across batches but also poses substantial challenges for large-scale manufacturing and quality control [20]. The wrinkled metal-coated thin-film sensing material was proposed to address the issue of a sudden increase in surface resistance caused by cracks on the surface of the aforementioned two materials under strain conditions, thereby ensuring the applicability of the sensing material under large strains. However, the contraction of the flexible substrate is irregular, resulting in randomly generated disordered wrinkles on the surface [21,22]. Consequently, the performance of this wrinkled metal-coated thin-film sensing material suffers from instability and irreproducibility in fabrication, making it difficult to produce on a large scale and apply in practice.

In addition, the emerging gel fiber, MXene-based and organic/inorganic hybrid stretchable sensing systems also have inherent defects in engineering applications: gel-based sensors generally have insufficient mechanical strength and poor long-term stability under cyclic loading, and are prone to swelling and failure in humid and corrosive environments [9]; MXene-based materials are prone to oxidative degradation in ambient atmosphere, resulting in irreversible attenuation of sensing performance, which limits their long-term service life [10]; organic/inorganic hybrid electrodes usually face the problem of conductive performance degradation under large strain, and their environmental corrosion resistance is difficult to meet the requirements of long-term outdoor structural health monitoring [11]. Therefore, it is urgent to develop a strain-sensing material that can simultaneously solve the problems of easy fracture of metal films, poor batch reproducibility of nanowire and wrinkled film systems, and insufficient environmental stability and mechanical durability.

This paper presents the development of a nanoscale patterned strain-resistive film sensing material based on nanofabrication technology. Different from the existing continuous metal film, random nanowire and disordered wrinkled film systems, the customized nanoscale patterned structure of the conductive layer can fundamentally avoid the strain localization and necking effects of the metal film under external load, thus solving the core problem of conductivity degradation and easy fracture of traditional metal film sensors under deformation. Meanwhile, the standardized nanofabrication process ensures the controllable preparation of the patterned structure, which effectively solves the problem of poor batch reproducibility of random nanowire and disordered wrinkled film systems, and is conducive to large-scale manufacturing and quality control. On this basis, the influence of environmental temperature on the sensing performance of the patterned strain-resistive film is systematically investigated, and the impact of environmental factors on the surface resistance of the sensing material is analyzed. Through targeted encapsulation technology, the degradation of conductive performance due to corrosive environments during long-term monitoring is effectively mitigated, thereby further enhancing the durability and environmental applicability of the sensor. This work provides a new solution for the development of strain sensors with high durability, excellent corrosion resistance and good batch reproducibility, which has broad application prospects in the field of long-term structural health monitoring under harsh environments.

## 2. Materials and Methods

Polydimethylsiloxane (PDMS) precursor and its curing agent were obtained from Dow Corning SYLGARD (Midland, MI, USA). The silver target employed for thin-film deposition had a purity of 99.99%. The master mold, made of SiO_2_, featured a periodic line array with a pitch of 200 nm, a line width of 100 nm, and a depth of 200 nm. While patterns with smaller periods and higher aspect ratios are generally more favorable for enhancing the mechanical stability of structured devices, the corresponding imprinting and demolding steps become increasingly challenging in nanoimprint lithography [23,24]. In addition, the sensing material fabricated in this work possesses a PDMS/Ag/PDMS sandwich structure. Owing to the different thermal expansion coefficients of the functional layers, temperature changes during the forming process can introduce additional strain [25]. Lowering the fabrication temperature and increasing the material thickness help alleviate this adverse effect. However, an excessively thin metal film and an overly small grating period will degrade the continuity and resistance stability of the Ag layer. Therefore, an Ag film thickness of 100 nm, a grating period of 200 nm, and a PDMS curing temperature of 70 °C are chosen in this study to minimize the above unfavorable effects.

For material processing and characterization, a vacuum drying oven (Lange, IPC-25, Jiangsu, China) was used to cure the PDMS polymer, while the metal layer was deposited via vacuum thermal evaporation (Vnano, VZZ-300, Beijing, China). Surface morphology was examined using scanning electron microscopy (SEM; Hitachi, S-4800, Tokyo, Japan). Electrical resistance measurements of the strain sensor were conducted with a multimeter (UNI-T, UT890C+, Guangdong, China).

**Figure 1:**
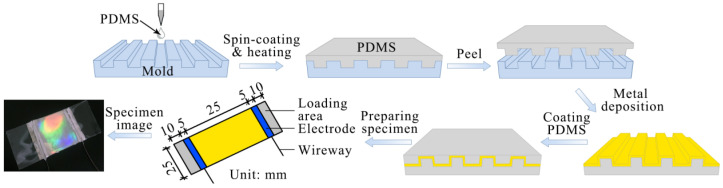
Schematic fabrication process of patterned PDMS/Ag/PDMS strain-sensing materials.

The complete fabrication workflow for the patterned polymer/metal/polymer device intended for sensing applications is schematically illustrated in Figure 1. Initially, the PDMS elastic layer, prepared at a 10:1 mixing ratio, was spin-coated onto the patterned SiO_2_ mold. Curing was subsequently performed in the vacuum drying oven under a pressure of 25 bar at 70 °C for 4 h. Following demolding, a 100 nm-thick Ag thin film was deposited onto the structured PDMS substrate under optimized conditions, specifically employing a deposition current of 170 A while maintaining the substrate on a cooling plate to regulate temperature. Electrical connections were then established by attaching copper wires to the Ag film using silver paste, with the opposite wire ends linked to the multimeter. The extremities of the specimen were designated as loading zones. To finalize the device, an additional PDMS encapsulation layer was spin-coated and cured over the patterned surface, serving as a protective barrier against silver oxidation and potential interfacial degradation. The resulting PDMS/Ag/PDMS strain sensor is depicted in the corresponding photograph within Figure 1.

## 3. Results and Discussion

### 3.1. Morphology of the Patterned Film

**Figure 2:**
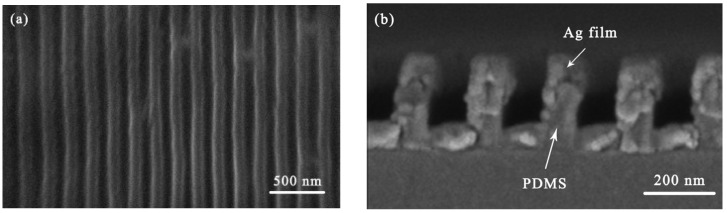
The SEM images of the patterned Ag/PDMS sensing material (**a**) Top view, (**b**) Sectional view.

Figure 2 presents the surface scanning electron microscopy (SEM) image of the fabricated patterned metallic Ag strain-sensing material. The image clearly reveals striped grating patterns with complete, uniformly distributed, and continuous surface features. Figure 2b shows the cross-sectional SEM image of the patterned metallic Ag strain-sensing material. The arrows in the image indicate the metallic silver thin film and the PDMS polymer. The SEM micrograph demonstrates that the surface layer consists of the deposited metallic Ag film, underlain by a PDMS polymeric substrate. From the cross-sectional view, the grating period is measured to be 200 nm, while the thickness of the surface-deposited metallic Ag film is approximately 100 nm, with the grating height reaching about 170 nm. Due to the bottom-up vertical deposition process in vacuum evaporation, the metal layer thickness on the PDMS sidewalls is thinner compared to the top and bottom surfaces. The cross-sectional details further confirm that the pattern from the SiO_2_ template is completely transferred to the PDMS substrate surface, and the metallic Ag film fully covers the PDMS surface to form a continuous metallic conductive film.

### 3.2. Effect of Film Length on Conductivity of the Sensor

**Figure 3:**
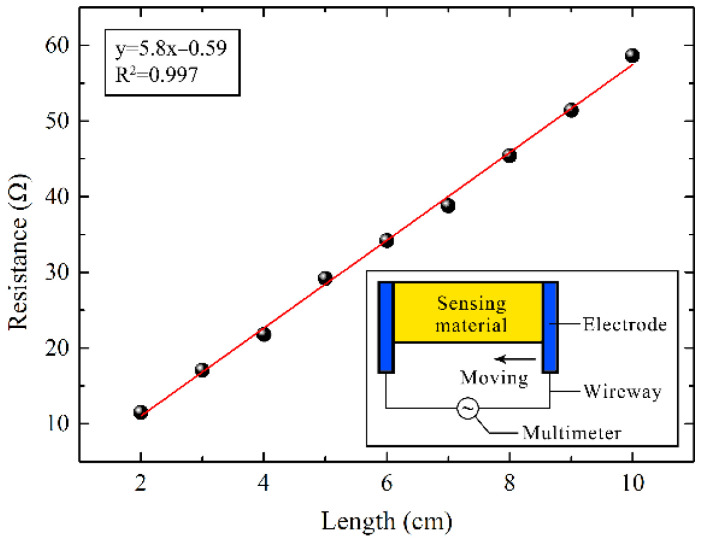
Schematic of measuring the relationship between resistance and length of strain sensor.

The dimensions of the sensing material must be tailored to the structural changes to be monitored; therefore, it is essential to analyze the influence of size on sensing performance based on specific application requirements. As shown in Figure 3, the resistance of sensors with widths ranging from 2 cm to 10 cm was measured using a multimeter. The points are the experimental data, and the red line is the linear fitting line. It can be observed from Figure 3 that the resistance of the sensor is proportional to the length of the thin film, with a fitted correlation coefficient of 0.997. The sensor can be regarded as an assembly of conductive thin films. As the length of the conductive film increases, the total resistance contributed by the micro-resistances also increases. This result further demonstrates the uniformity of the film. Consequently, the resistance of a conductive film of any length can be calculated based on the resistance of a film with a specific length, which is essential for the practical application of strain sensors.

### 3.3. Effect of Temperature on Conductivity of the Sensor

**Figure 4:**
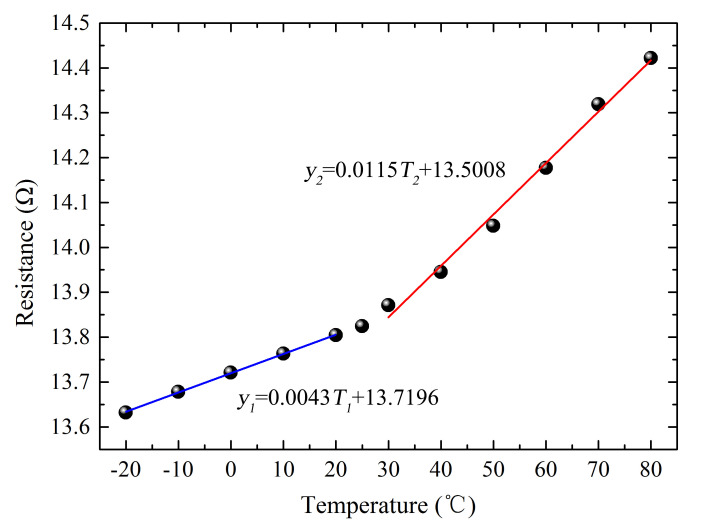
Variation in Resistance of the Strain Sensor at Different Temperatures.

Figure 4 presents the resistance values of the sensor measured at different temperatures. The resistance was recorded after placing the sensor in a vacuum oven or refrigerator set to predetermined temperatures for one hour. As illustrated in Figure 4, when the temperature reaches 80 °C, the resistance increases to 14.42 Ω; at −20 °C, the resistance decreases to 13.63 Ω. This temperature range is sufficient to accommodate most practical application environments. From the measured data, it can be observed that within the ranges of 30 °C to 80 °C and −20 °C to 20 °C, the relationship between resistance and temperature is approximately linear. An inflection point in resistance is observed at around 25 °C. The resistance of pure metallic materials usually varies linearly with temperature. However, for the multilayer thin-film sensor in this work, the different thermal expansion coefficients of each functional layer will induce mismatched mechanical strain under temperature changes. Specifically, PDMS undergoes significant thermal expansion as temperature rises, which introduces extra mechanical strain and leads to the non-linear resistance variation in the sensor. When the temperature drops below this point, the resistance decreases and gradually stabilizes. As the temperature rises, atomic thermal motion intensifies, leading to increased disorder in electron movement [26]. Furthermore, the rate of change in resistance is higher at elevated temperatures compared to lower temperatures.

### 3.4. Effect of Tensile Properties

**Figure 5:**
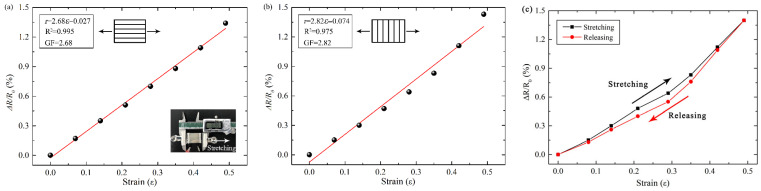
Tensile performance and hysteresis of the patterned strain sensors. (**a**) Parallel stretching to the pattern; (**b**) Perpendicular stretching to the pattern; (**c**) Hysteresis test.

Tensile performance is the most commonly evaluated functional characteristic in sensing measurements, and the tensile behavior of the sensing material was examined after cyclic loading as shown in Figure 5. The loading zone of the sensing material was fixed using a vernier caliper, and variations in resistance under different strain conditions were simulated by adjusting the distance of the right caliper. The stability of the patterned strain-sensing film under cyclic loading was characterized. The film was subjected to 2000 stretching–releasing cycles with a maximum strain of 0.5. Initially, the baseline resistance of the film was 13.8 Ω. As the number of cycles increased to approximately 100, the baseline resistance gradually rose and finally stabilized at 15.3 Ω. Meanwhile, the resistance change rate (ΔR/R_0_) at the maximum strain of 0.5 increased slightly from 1.4 to 1.46 and then remained stable. This initial growth followed by subsequent stabilization can be attributed to the initiation of microcracks in the metallic thin film under tensile stress, as well as the gradual saturation and stabilization of these cracks. Furthermore, the patterned structure mitigates the further propagation of these microcracks. The stable performance after 2000 cycles demonstrates that the patterned Ag strain-sensing film possesses excellent long-term operational stability, which meets the requirements for practical applications. Notably, a pre-stretching process of 100 cycles can be applied to eliminate the initial resistance drift of the sensor, thereby improving the accuracy of practical measurements.

Due to the directional nature of the patterned sensing material, tensile performance was analyzed for two distinct loading orientations. As shown in Figure 5a,b, both the strain and the relative resistance change exhibited a linear relationship, with high correlation coefficients in each case. When the stretching direction was parallel to the pattern, the rate of change was slightly lower than that observed when the stretching direction was perpendicular to the pattern. The maximum strain applied during testing was 0.5, which is sufficient to accommodate most practical application scenarios.

To investigate the hysteresis and reversibility of the patterned thin-film strain-sensing material, the sensing material was stretched to a strain of 0.5 and then recovered to its initial state within a total duration of 10 s, with the surface resistance change rate calculated, as shown in Figure 5c. The black curve represents the resistance change rate during the stretching process, while the red curve corresponds to that during the recovery process. As observed from the figure, during the recovery of the patterned Ag strain-sensing material from a strain of 0.5 back to the initial state, the sensing material exhibits a certain degree of hysteresis, and the recovery curve does not overlap with the stretching curve. In the initial stage of strain recovery, the hysteresis of the sensing material is relatively small. As the strain continues to recover, the hysteresis of the resistance change rate shows a trend of first increasing and then decreasing, with the maximum hysteresis range occurring at a strain of 0.3. When the sensing material returns to its initial state with zero strain, the resistance gradually recovers to its initial value. These results demonstrate that the patterned Ag strain-sensing material possesses high reversibility and low sensing hysteresis during the stretching and releasing processes.

### 3.5. Anti-Corrosion Property

In practical applications, sensing materials are often exposed to environmental factors such as humidity and corrosion. In this section, the performance of the fabricated sensor under high-salinity conditions was investigated. A saline solution with a salt concentration exceeding that of seawater (3.5%) was prepared by thoroughly mixing 100 g of deionized water with 5 g of sodium chloride until complete dissolution. The sensor was subsequently immersed in the saline solution. To prevent corrosion of the copper wires in the saline environment, they were isolated from the solution using a plastic film, thereby preserving the electrical conductivity of the sensor. A control group was set up in this experiment to investigate the effect of the PDMS protective film on the stability of the sensor: R_1_ refers to the resistance of the sensor with a surface PDMS protective film, while R_2_ represents the resistance of the silver thin-film sensor without a surface PDMS protective film. The resistances of the two sensing materials were measured at different immersion durations, and the results are summarized in Table 1.

It can be observed from Table 1 that the initial resistance of the sensor with the PDMS protective film (R_1_) was 13.65 Ω, and after 60 days of immersion in the saline solution, the resistance remained stable at approximately 13.6 Ω. In contrast, the initial resistance of the silver thin-film sensor without the PDMS protective film (R_2_) was 13.71 Ω. With the extension of immersion time, the resistance of R_2_ showed a significant upward trend, increasing to 32.61 Ω after 60 days, indicating obvious corrosion and aging phenomena. The stability of R_1_ is attributed to the protective effect of the PDMS film, which can block the direct contact between the conductive layer and the saline solution, thereby avoiding chemical reactions that would impair the electrical conductivity of the sensor. In addition, the low surface energy of the PDMS layer endows the composite film with hydrophobicity and corrosion resistance. In contrast, due to the lack of such protection, the silver thin film of R_2_ is in direct contact with the high-salinity solution, leading to oxidation, grain boundary corrosion and other reactions, which results in a continuous increase in resistance. The comparison between the two groups further demonstrates that the sensor with the PDMS protective film designed in this study is suitable for practical applications in high-salinity and humid environments.

### 3.6. Micro-Strain Monitoring

**Figure 6:**
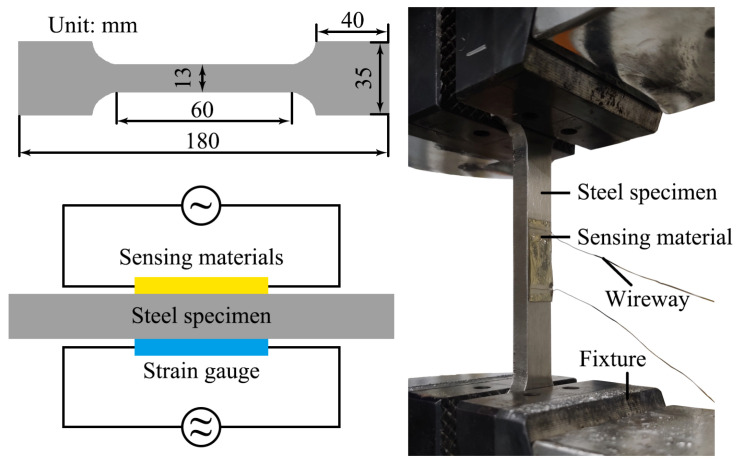
Photograph of micro-strain monitoring experiment instrument.

Figure 6 presents a photograph of the experimental setup used for micro-strain monitoring. The strain sensor and a commercial strain gauge were both attached to the surface of a tensile specimen, which was then subjected to tensile loading using a universal testing machine. The stretching speed of the universal testing machine was controlled at 0.2 mm/min. Electrical conductivity was measured by connecting a resistance meter via copper wires to evaluate the applicability of the sensing material. The flexible sensing film possesses excellent flexibility and can be tailored to the size and shape of the monitored structure. In this work, the sensor dimensions were adjusted according to the actual size of the tested steel specimen, resulting in the difference between the current measurement data and the previous results.

**Figure 7:**
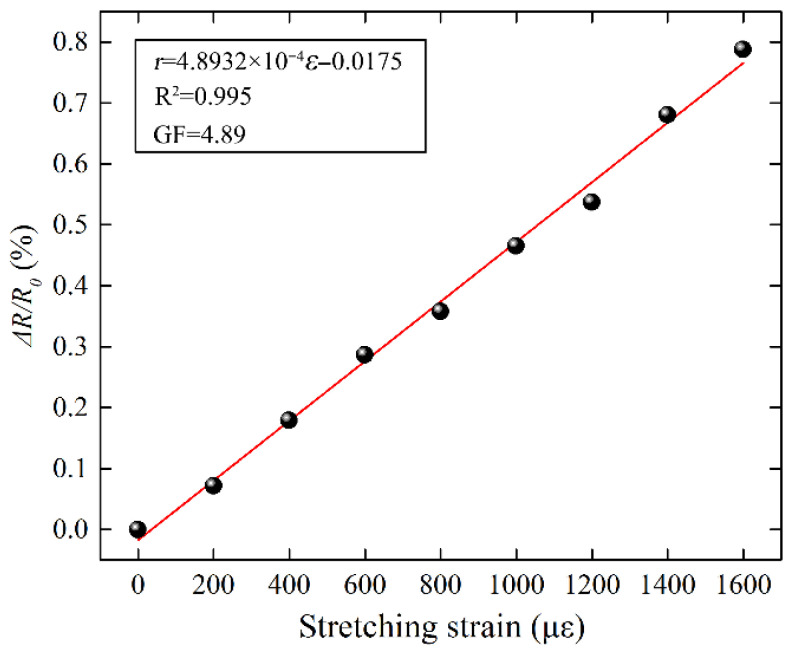
Real-time monitoring of relationship between micro stretching strain and ΔR/R0 on the steel sheet.

Figure 7 shows the relative resistance change (Δ*R/R*_0_) under various micro-strains ranging from 0 to 1600 με. It can be observed that Δ*R/R*_0_ increases linearly with increasing tensile strain, exhibiting a high correlation coefficient of 0.995. When the strain reached 1600 με, the ΔR/R0 of the sensor increased to 0.77%, corresponding to a gauge factor (GF) of 4.89, where GF is defined as (Δ*R/R*_0_)/ε, with ε representing the applied strain. In comparison, commonly used flexible sensing materials such as carbon nanotubes typically exhibit GF values of 0.57 and 1.73 under different conditions, both of which are substantially lower than that of the strain sensor fabricated in this work. Given its excellent linearity and high sensitivity, the proposed flexible strain sensor shows significant promise for potential applications in real-time strain monitoring.

## 4. Conclusions

This paper presents the fabrication of a flexible strain sensor based on a sandwich-structured, patterned metal film with high durability and corrosion resistance. The effects of the sensor film’s dimensions and ambient temperature on its electrical conductivity were systematically investigated, confirming its suitability for applications involving varying scales and environmental conditions. The patterned sensing material exhibited exceptional durability, maintaining stable performance even after 500 stretching cycles. Furthermore, the sensing material remained effective in saline and high-humidity environments. When mounted on a steel plate for real-time micro-strain detection during tensile testing, the sensor demonstrated a highly linear relationship between the relative resistance change (Δ*R/R*_0_) and the applied strain, along with high sensitivity. The outstanding durability and corrosion resistance of this conductive film underscore its significant potential for use as a flexible strain sensor.

## Figures and Tables

**Table 1:** Variation in resistance with immersion time.

Time	0 d	15 d	30 d	45 d	60 d
R_1_ (Ω)	13.65	13.70	13.67	13.62	13.68
R_2_ (Ω)	13.71	16.12	19.33	25.27	32.61

## Data Availability

The original contributions presented in this study are included in the article. Further inquiries can be directed to the corresponding author.

## Abbreviations

The following abbreviations are used in this manuscript:

SHM	Structural health monitoring
PDMS	Polydimethylsiloxane
SEM	Scanning electron microscopy
GF	Gauge factor
